# Assessment of the cellular localisation of the annexin A2/S100A10 complex in human placenta

**DOI:** 10.1007/s10735-018-9791-2

**Published:** 2018-08-24

**Authors:** Seham A Abd El-Aleem, Lodewijk V. Dekker

**Affiliations:** 1 School of Pharmacy, Centre for Biomolecular Sciences, University of Nottingham, Nottingham, NG7 2RD UK; 2 Present Address: Department of Histology, Minia Faculty of Medicine, Minia, Egypt

**Keywords:** Protein–protein interactions, Proximity ligation assay, Annexin A2, S100A10, Placenta, Trophoblast

## Abstract

The AnxA2/S100A10 complex has been implicated in various placental functions but although the localisation of these proteins individually has been studied, there is no information about the localisation of their complex in situ at the cellular level. Using the proximity ligation technique, we have investigated the in situ localisation of AnxA2/S100A10 complex in the placenta and have compared this with the location patterns of the individual proteins. High levels of expression of AnxA2/S100A10 complexes were observed in the amniotic membrane and in blood vessel endothelial cells. Lower levels were detected in the brush border area of the syncytium and in the trophoblasts. Immunohistochemical analysis of AnxA2 and S100A10 individually revealed broadly similar patterns of localisation. The brush border staining pattern suggests that in this location at least some of the AnxA2 is not in complex with S100A10. The formal location of the AnxA2/S100A10 complex is compatible with a role in cell–cell interaction, intracellular transport and secretory processes and regulation of cell surface proteases, implying contributions to membrane integrity, nutrient exchange, placentation and vascular remodelling in different parts of the placenta. Future applications will allow specific assessment of the association of the complex with pathophysiological disorders.

## Introduction

Although the placenta is anatomically variable across mammalian species, a core transcriptome of around 115 genes can be identified that are critical to the function of the placenta across species (Armstrong et al. 2017). Predominant amongst these are members of the annexin family, AnxA2, AnxA1 and AnxA5, arguing for the evolutionary importance of annexins (Armstrong et al. 2017). Annexins are also known as ‘placental anti-coagulant proteins’ and they have been estimated to constitute up to 2% of the total placental membrane proteins (Buhl et al. 1991).

AnxA2 appears to be a predominant factor in the core placental transcriptome (Armstrong et al. 2017). It exists in multiple molecular forms, including a monomeric form and a heterotetrameric complex of two molecules of AnxA2 and two molecules of S100A10, a member of the S100 family (Rety et al. 1999). S100A10 is also present in the core transcriptome mentioned above, suggesting that the complex may be a relevant entity in the placenta. The heterotetrameric complex will be referred to as AnxA2/S100A10 complex hereafter.

AnxA2 contains a core Ca^2+^ and phospholipid binding domain and a small N-terminal tail domain. This tail domain consists of 30 amino acids of which the first 14 residues constitute the binding site for S100A10 allowing formation of the AnxA2/S100A10 complex (Liu et al. 2015). Both the AnxA2 monomer and the AnxA2/S100A10 complex have membrane binding functions, although as part of the complex AnxA2 has a lower Ca^2+^ requirement for membrane association and as such has a different intracellular distribution compared with monomeric AnxA2 (Monastyrskaya et al. 2007; Powell and Glenney 1987). The membrane binding function of AnxA2, alone or in complex, may actuate roles in endo or exocytosis and/or the regulation of intracellular transport of various ion channels and transporters (Gerke et al. 2005; Liu et al. 2015).

Inside the cell, the AnxA2/S100A10 complex localises to the cytosolic surface of the plasma membrane and can associate with the submembranous cytoskeleton (Thiel et al. 1992). The AnxA2/S100A10 complex displays F-actin binding and bundling activity at physiological Ca^2+^ concentrations (Ikebuchi and Waisman 1990; Jones et al. 1992) and has also been characterised as a tonic inhibitor of cytosolic phospholipase A2; phosphorylation at position 727 is required to release the AnxA2/S100A10 complex, resulting in enzyme disinhibition and generation of the prostaglandin biosynthesis precursor arachidonic acid (Aarli et al. 1997; Bennett et al. 1994; Tian et al. 2008). Formation of the complex and resulting membrane association is also a prerequisite for tyrosine phosphorylation and externalisation of the complex, such that it becomes localised on the extracellular face of the membrane (Deora et al. 2004; Zheng et al. 2011). These surface AnxA2/S100A10 complexes have been implicated in activation of surface proteases and matrix metalloproteinases and regulation of extracellular matrix invasion (Hajjar and Hamel 1990; Ling et al. 2004; Surette et al. 2011) as well as cell–cell interactions (Lee et al. 2008, 2004; Myrvang et al. 2013).

Thus AnxA2 may have several roles in the placenta, including regulation of the phospholipase A2 system and the production of placental prostaglandins, the translocation of ion channels and transporters that are involved in nutrient exchange at the foeto–maternal interface, epithelial cell–cell interactions and in the regulation of extracellular proteases, which may in turn be important in cell migration and establishment of essential placental structures as well as prevention of blood clotting (Pfarrer 2006; Xin et al. 2012).

Localisation studies revealed that AnxA2 localises to the amnion epithelium, trophoblast cells and blood vessel endothelial cells (Bogic et al. 1999; Sun et al. 1996). It was also detected in syncytiotrophoblasts (Xin et al. 2012), on the surface of syncytiotrophoblasts (Kristoffersen and Matre 1996) and in brush border vesicles in the microvillar region of syncytiotrophoblasts (Kaczan-Bourgois et al. 1996) although one report indicated it was absent from the latter cells (Sun et al. 1996). Like AnxA2, S100A10 was detected in the brush border vesicles of the syncytiotrophoblasts (Kaczan-Bourgois et al. 1996). Quantification by western blotting suggested that the level of S100A10 is lower than that of AnxA2, indicating that some of the AnxA2 population existed in its monomeric form (Kaczan-Bourgois et al. 1996). This is compatible with models that suggest that S100A10 only exists in complex with AnxA2 and that the amount of S100A10 determines the amount of complex (He et al. 2008). S100A10 is also present on the surface of the syncytiotrophoblast microvillous plasma membrane and it was suggested that at this site, it forms the heterotetramer with AnxA2 (Kristoffersen and Matre 1996).

Our understanding of the role of the AnxA2/S100A10 complex in the placenta is limited by the fact that we do not have accurate information on its precise location. All localisation studies done so far assessed the two proteins individually and this data has been used to extrapolate the location of the complex. Direct data on the location of the AnxA2/S100A10 complex in situ in the placenta is thus highly desirable. The recent development of the proximity ligation technology has allowed the location of protein complexes in situ. Here we assessed the in situ location of AnxA2/S100A10 complexes in placental and foetal membranes using proximity ligation. We provide a comprehensive mapping of the complex and of the individual proteins on parallel placental sections and discuss possible roles of the proteins in placenta function.

## Experimental

### Materials

6 µm formalin-fixed paraffin-embedded placenta tissue sections were cut from 8 separate tissue blocks obtained from two collections (the Department of Histopathology collection and the University of Nottingham tissue bank facility at the University of Nottingham NHS trust). Tissues were obtained anonymously after consent from the donors. Antibodies were obtained from BD Bioscience (Purified Mouse anti-AnxA2, Catalogue number: 610068 and Purified mouse anti-S100A10, Catalogue number: 610070) and from Thermo Fisher (Polyclonal rabbit anti S100A10, Catalogue number: PA5-26100). Antibody specificity had been demonstrated previously using Western blotting (Myrvang et al. 2013). Immunohistochemistry detection kit was obtained from Leica (Novolink Polymer Detection System, Catalogue number: RE7150-K). The Duolink II in situ brightfield kit was obtained from Sigma Aldrich (DUO92012100RXN).

### Immunohistochemical staining for AnxA2 and S100A10

Sections were dewaxed in xylene and hydrated through alcohol gradient. Antigen retrieval was performed in 0.01 M sodium citrate buffer (pH 6) for 20 min in a microwave at 750 W. Endogenous peroxidase activities was blocked using hydrogen peroxide for 10 min. Primary antibodies; purifies mouse anti-AnxA2 (1:300), purified mouse anti-S100A10 (1:250) were applied to the tissue overnight at 4 °C. The staining was completed using Novolink kits and reagents were prepared and used according to the manufacturer’s instructions. Briefly, sections were incubated with post-primary (30 min at room temperature) followed by polymer (30 min at room temperature). Reactions were developed with 3,3′-diaminobenzidine. Sections were then counterstained with haematoxylin and dehydrated prior to mounting with DPX and cover slipped for microscopic examination. Multiple staining runs were performed, with each run including a negative control in which primary antibodies were replaced with normal serum of the host species of the secondary antibody. Positive signals appear as brown staining on the sections with the counterstaining in blue.

### Detection of the AnxA2/S100A10 complex

A Duolink II in situ brightfield proximity ligation kit was used to determine the AnxA2/S100A10 interaction on sections. Placenta sections were dewaxed in xylene and hydrated through an alcohol gradient. Antigen retrieval was performed in 0.01 M sodium citrate buffer (pH 6) for 20 min in a microwave at 750 W. The in situ proximity ligation staining was performed using reagents provided in a Duolink II in situ brightfield kit prepared according to the manufacturer’s instructions. Briefly, after blocking endogenous peroxidase (10 min at room temperature) and non-specific protein binding (1 h at room temperature), sections were incubated overnight at 4 °C with the primary antibody pair. Sections were then incubated with the probe mix (mouse PLUS and rabbit MINUS secondary in situ proximity ligation antibodies) for 1 h at 37 °C. Subsequently, ligation (1 h at 37 °C) and amplification (2 h at 37 °C) reactions were carried out on all slides. The complex was visualized by reaction with HRP detection brightfield solution (1 h at room temperature) followed by the substrate mix for 7 min at 20 °C. Sections were then counterstained for 2 min in haematoxylin. Following 10 min wash in running tap water, sections were dehydrated in grades of ethanol 96% then 99% 2 min each before being transferred to xylene. Sections were mounted by using in situ brightfield mounting medium, a xylene based mounting medium and then cover slipped for microscopic examination. Multiple staining runs were performed, with each run including a negative control in which primary antibodies were replaced with antibody diluent solution. Positive signals appear as brown/red dots with the counterstaining in blue.

### Microscopy and image capturing

Tissue sections were viewed by light microscopy (Nikon Eclipse). Images were digitally captured on a computerized image analysis system consisting of a colour analogue camera connected to a computer and processed in Adobe Photoshop.

## Results

Immunohistochemical staining was performed to study the cellular distribution of AnxA2 and S100A10 on parallel sections of paraffin embedded human placenta tissues. In situ proximity ligation was performed for detection of the AnxA2/S100A10 heterotetrameric complexes, at the cellular level. Consistent staining patterns were observed between placental blocks and between staining runs.

### Detection of AnxA2, S100A10 and the AnxA2/S100A10 complex in the amniotic membranes and chorionic plate

**Fig. 1:**
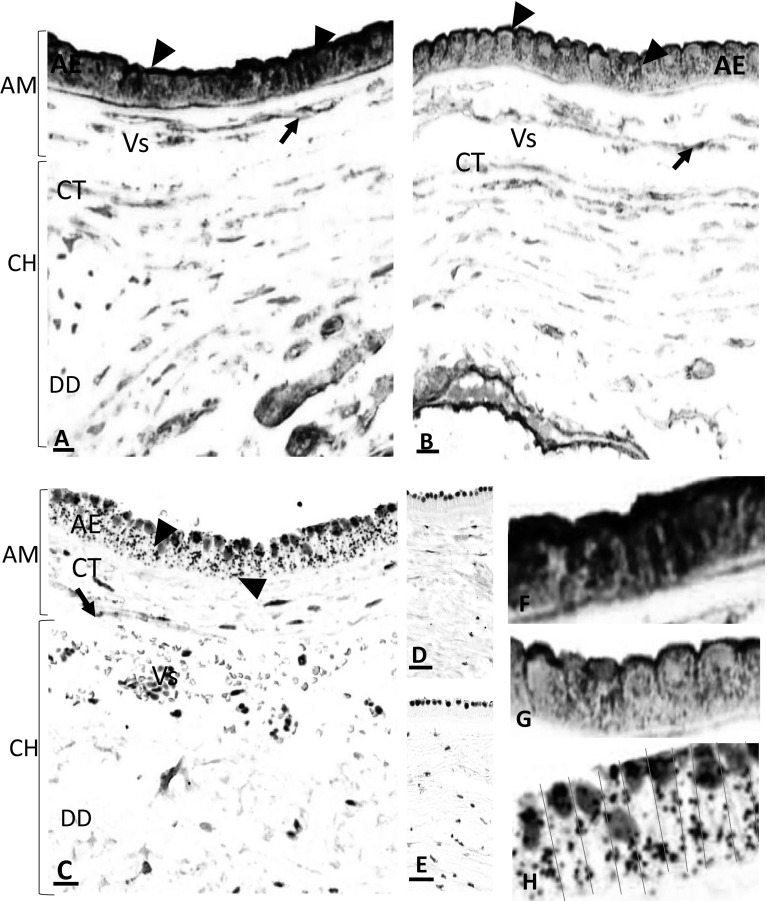
Location of AnxA2, S100A10 and the AnxA2/S100A10 complex in the amniotic membranes. Parallel sections from the same placental block were stained for AnxA2 (**a, f**), S100A10 (**b, g**) or the AnxA2/S100A10 complex (**c, h**). AnxA2 and S100A10 staining is in brown, the proximity ligation signal is represented as brown dots and haematoxylin counterstaining is in blue. Representative images are shown. **d, e** are negative controls showing absence of the positive signals. The amniotic epithelium (AE), amniotic connective tissue (CT) containing blood vessels (Vs) and their endothelial lining (arrows), Chorion (CH) and Decidua (DD) are indicated. Arrowheads indicate apical and junctional staining of epithelial cells. Scale bars: **a**–**c** 25 µm. **d, e** 5 µm

Examination of the amniotic membranes and underlying structures revealed very strong staining for AnxA2 in the amniotic epithelium as well as in the amniotic connective tissues, mainly in the vascular endothelial lining and few scattered connective tissue mesenchymal cells (Fig. 1a). Strong immunoreactivity was observed at the amniotic epithelial cell membrane both apically as well as at the junctional intercellular face (Fig. 1a, f). S100A10 immunoreactivity showed similar patterns of localisation (Fig. 1b, g). The negative control did not show staining (Fig. 1d).

**Fig. 2:**
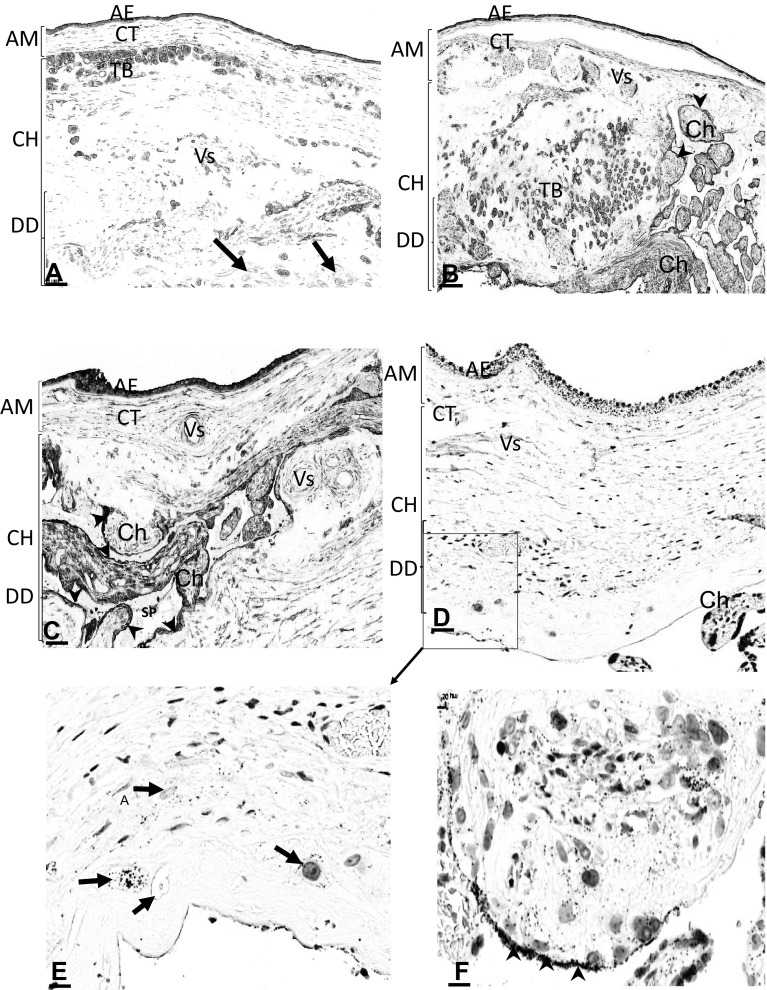
Location of AnxA2, S100A10 and the AnxA2/S100A10 complex in the foetal part and chorionic plate of the placenta. Sections from the same placental block were stained for AnxA2 (**a, b**), S100A10 (**c**) or the AnxA2/S100A10 complex (**d**–**f**). Representative images of the foetal side of the placenta are shown in low magnification. Amniotic (AM), chorionic (CH) and decidual (DD) parts are indicated, including the amniotic epithelium (AE), amniotic connective tissue (CT), chorionic trophoblasts (TB), chorionic villi (Ch), decidual cells (arrows in **a**), syncytiotrophoblast layers (arrowheads in **b, c** and **f**) and spiral vessels (SP in **c**). **e, f** show higher magnifications of proximity ligation stained sections indicating expression of the complex in the trophoblasts (arrows; **e**) and at the marginal surface of the syncytiotrophoblast (arrowheads; **f**). Scale bars: **a, b** 70 µm, **c** 60 µm, **d** 50 µm, **e** 20 µm, **f** 10 µm. Staining is as described in the legend of Fig. 1

To assess whether these proteins form a complex in situ, we performed proximity ligation assays on parallel sections of the placenta. Figure 1c shows the staining pattern whist Fig. 1e shows the negative section without primary antibodies. Specific staining (apparent as brown/red dots) was observed in the same areas as the immunoreactivity for individual markers, albeit that the signal appeared less intense as these markers (Fig. 1c, h). Thus, a strong proximity ligation signal was observed in the amniotic epithelium cell layer (Figs. 1c, 2d), indicating that these proteins do indeed exist as a complex in this location. Like the signal for the individual AnxA2 and S100A10 markers, the proximity ligation signal was observed at the apical side of the epithelial cells, and in columns which may reflect location at the intercellular interface (Fig. 1h). Negative control sections did not show any staining (Fig. 1e) confirming that the in situ proximity ligation staining was specific for the primary antibodies used.

Examination of the chorionic plate indicated the presence of AnxA2 in the chorionic trophoblasts, vascular endothelial lining and chorionic villi. At the amnion-chorionic junction, AnxA2-expressing trophoblasts were seen just underneath the amniotic membrane (Fig. 2a). Dense AnxA2 staining was observed in migrating trophoblasts invading the maternal decidua (Fig. 2b). Budding and floating chorionic villi near the chorionic plate showed dense AnxA2 immunoreactivity in the syncytiotrophoblasts (Fig. 2b). The maternal decidua cells themselves show weak AnxA2 immunoreactivity (Fig. 2a). In the chorionic region, S100A10 immunoreactivity was seen in most of the chorionic villi (Fig. 2c) in the chorionic trophoblasts and vascular endothelial lining (Fig. 2c). The budding and floating chorionic villi nearby the chorionic plate showed S100A10 immunoreactivity in the syncytiotrophoblast (Fig. 2c). Maternal spiral arteries were seen invaded by chorionic villi, with strong S100A10 immunoreactivity being visible in the endothelial lining (Fig. 2c).

Similar to the pattern observed in the amniotic membrane, the staining for the AnxA2/S100A10 complex by proximity ligation on parallel sections of the chorionic plate appeared much less sensitive than the staining observed for the individual markers. Nevertheless, expression of the complex was seen in similar structures, in the chorionic trophoblasts and in chorionic villi (Fig. 2d, e). At higher magnification a budding anchored chorion showed dense expression of the complex at the marginal surface in the syncytiotrophoblast (Fig. 2f), similar to what was observed for S100A10 staining (Fig. 2c).

### Detection of AnxA2, S100A10 and the AnxA2/S100A10 complex in the chorion

**Fig. 3:**
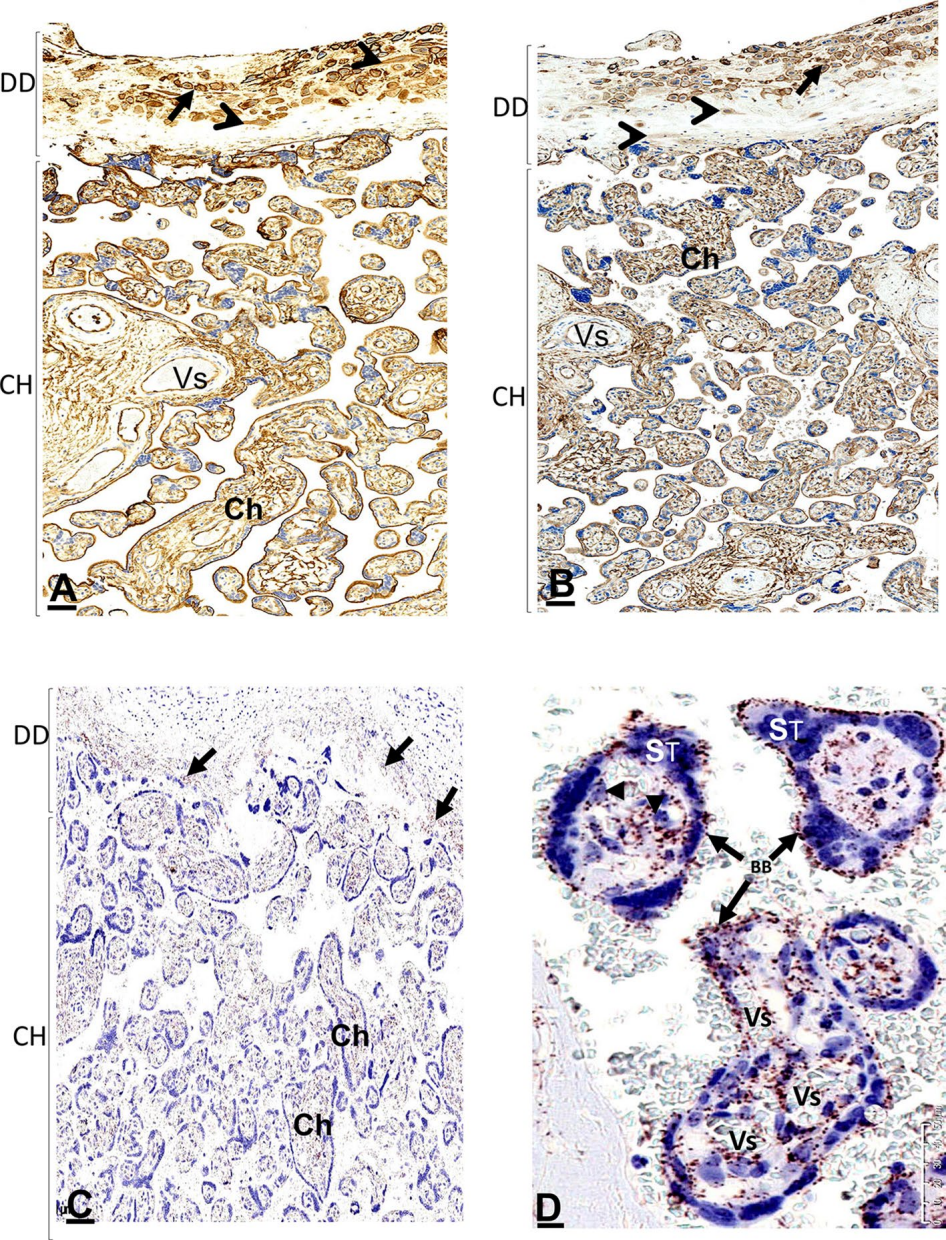
Location of AnxA2, S100A10 and the AnxA2/S100A10 complex in the decidual and in the chorionic parts of the placenta. Sections from the same placental block were stained for AnxA2 (**a**), S100A10 (**b**) or the AnxA2/S100A10 complex (**c, d**). Representative images are shown. The maternal part (decidual part, DD) is seen with immunoreactive cells identified as decidual cells (arrowheads) and trophoblasts (arrows). The chorion part (CH) is seen with many chorionic villi (Ch) and chorionic blood vessels (Vs). D is a higher magnification showing the detailed structure of a floating chorionic villi; brush border (BB), chorionic vessels (Vs), cytotrophoblasts (arrowheads), chorionic vessels (Vs) and outermost multinucleated syncytiotrophoblast layer (ST). Scale bars: **a**–**c** 50 µm; **d** 10 µm. Staining is as described in the legend of Fig. 1

Both AnxA2 and S100A10 were observed in the invading chorionic trophoblasts within the maternal decidua as well as in the junctional zone between the decidua and the chorion (Fig. 3). Anchored and floating chorionic villi of variable size with dense immunoreactivity were seen in between the chorionic plate and the decidua (Fig. 3a, b). Proximity ligation staining suggests that in these areas, the proteins exist in complex as the proximity ligation signal was also observed at the junctional zone and in chorionic villi (Fig. 3c). Within the chorionic villus the proximity ligation signal was seen in the foetal chorionic blood vessels, cytotrophoblast and the brush border area of the syncytiotrophoblast (Fig. 3d).

**Fig. 4:**
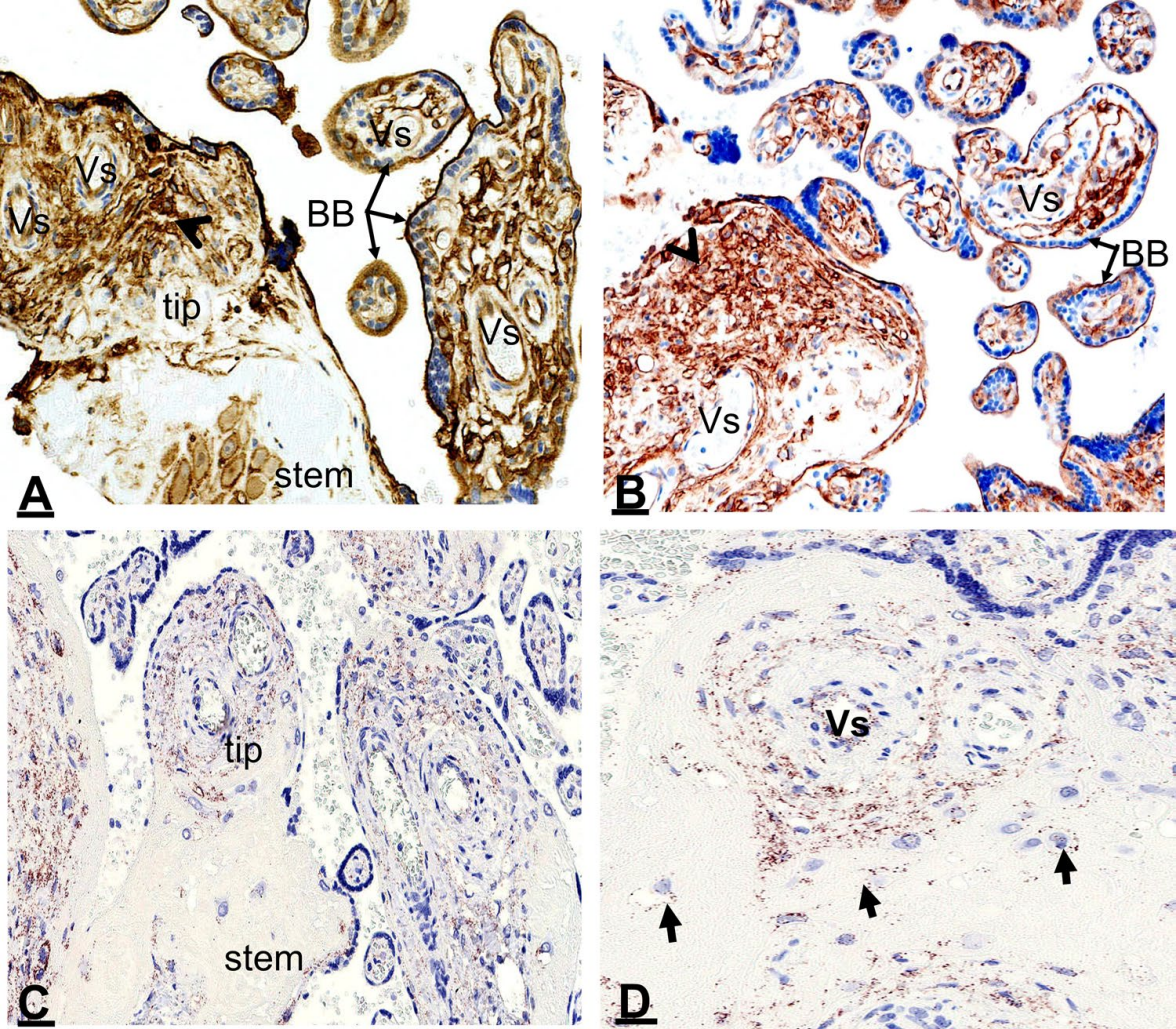
Location of AnxA2, S100A10 and the AnxA2/S100A10 complex in the chorionic villi. Sections from the same placental block representing the villus areas were stained for AnxA2 (**a**), S100A10 (**b**) or the AnxA2/S100A10 complex (**c, d**). The tip and stem of the villus, as well as chorionic blood vessels (Vs), brush borders (BB) and chorionic cells (arrowheads) are indicated. In D, remodelled mature chorionic vessel (Vs) showing expression of the complex in the vascular endothelial lining and vascular smooth muscle layers. In chorionic stroma the complex is seen in the cell membrane of chorionic cells (arrows). Scale bars: **a, d** 25 µm; **b, c** 40 µm. Staining is as described in the legend of Fig. 1

AnxA2 itself showed strong immunoreactivity in the brush border covering the villi (Fig. 4a) and in the chorionic connective tissue stroma core, mainly in the foetal vascular endothelial lining (Fig. 4a) as well as in scattered stromal cells which can be identified as cytotrophoblast and Hofbauer cells (Fig. 4a). The multinucleated syncytiotrophoblast layer covering the chorion showed a variation in expression from chorion to chorion usually limited to the outer membranous surface which is not distinguished from the brush border (Fig. 4a). S100A10 localisation showed a similar anatomical and cellular pattern to AnxA2 (Fig. 4b). The brush border staining of AnxA2 was more intense than endothelial staining inside the villi whilst the opposite appears to be the case for S100A10 in that the intensity of brush border staining appears lower than that inside the villi. This suggests that the relative amount of S100A10 in the brush border region is lower than AnxA2 and that some of the AnxA2 staining appears to represent the AnxA2 monomer. Proximity ligation staining clearly suggests that a complex between the two proteins is also present in the brush border (Fig. 3d). The complex can be observed in both anchored (Fig. 4c) and floating (Figs. 3d, 4d) chorionic villi. Inside the villi, it can be detected in the chorionic blood vessels and cells located at the tip with low expression in the stem whilst trophoblasts in the stem also show weak staining (Fig. 4c, d). In the chorionic villi, the complex was expressed in the syncytiotrophoblast layer mostly on the outer surface and the brush border (Fig. 3d). At higher magnification of a chorionic villous, the complex was seen in the foetal blood capillaries (Figs. 3d, 4d). In remodelled mature chorionic vessels, the complex was observed in the vascular lining and the vascular smooth muscle layers (Fig. 4d). In chorionic trophoblasts the complex was seen mainly in the cell membrane (Fig. 4d).

### Cellular and subcellular localisation of AnxA2, S100A10 and the AnxA2/S100A10 complex in trophoblasts

**Fig. 5:**
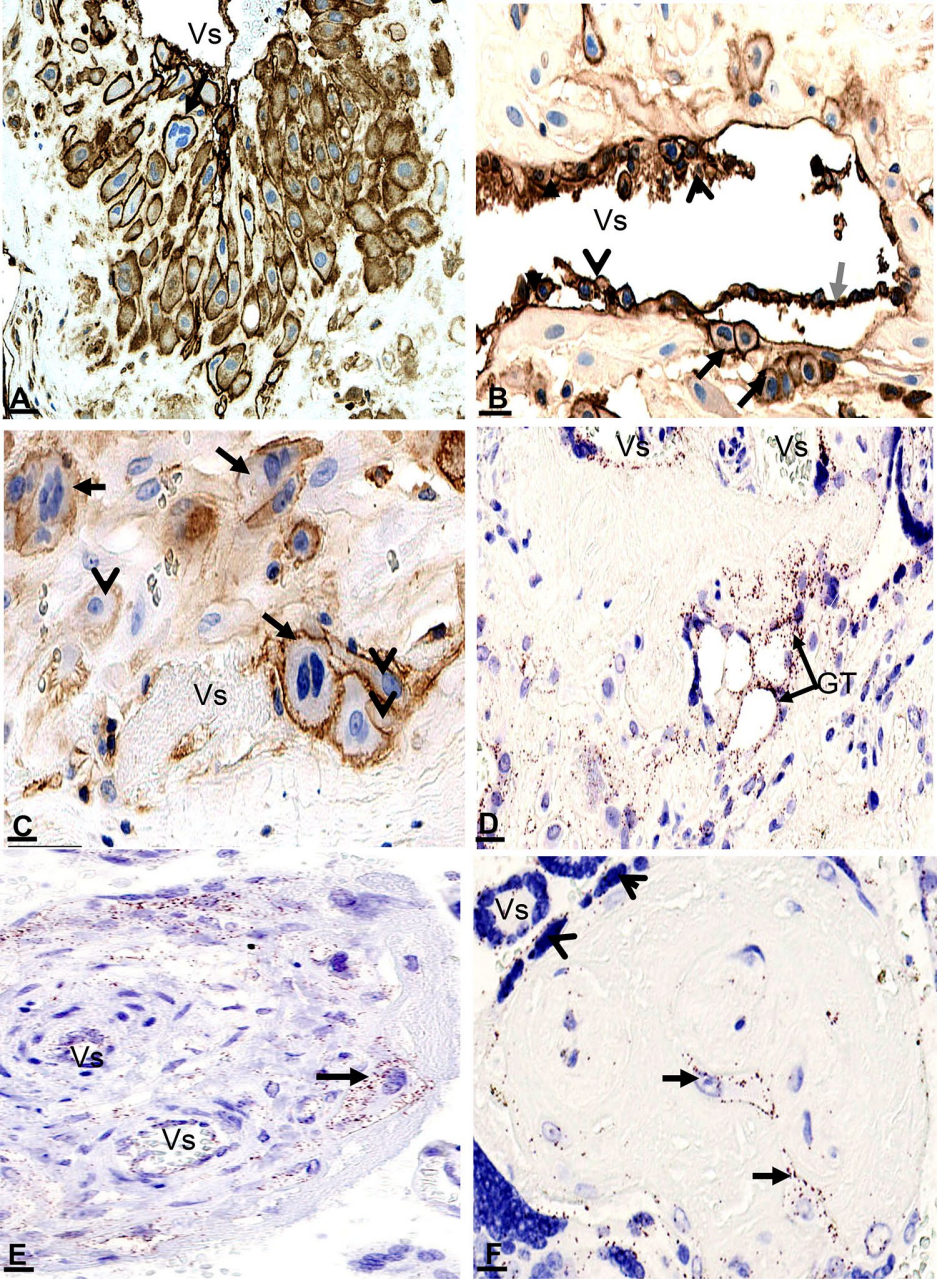
Cellular and subcellular localisation of AnxA2 (**a**) and S100A10 (**b, c**) and AnxA2/S100A10 complex (**d**–**f**) in the placenta. Enlargements of chorionic areas showing cell types and subcellular localisation of the complex in: **a** trophoblasts, including trophoblast giant cells (arrow) invading a maternal vessel (Vs) and forming the wall of the vessel. **b** A remodelling vessel with interstitial trophoblasts invading perivascular tissue (black arrows), disorganised vessel wall with detachment of the elastic lamina (grey arrow) and endovascular trophoblasts entered and incorporated into the vessel wall (arrowheads). **c** Trophoblasts (arrowheads) and trophoblast giant cells (arrows) residing along a vessel (Vs). **d** Trophoblast glycogen cells (GT). **e** Trophoblast giant cell within a chorionic villus with (Vs). **f** Hofbauer cells (arrows) and syncytiotrophoblast (arrowheads). Blood vessels (Vs) seen in **a**–**f** show endothelial cell expressions. Scale bars: **a** 30 µm; **b**–**f** 20 µm

The expression of AnxA2 and S100A10 in chorionic trophoblasts was very clear in this study and trophoblast behaviour could be easily followed on the basis of the staining patterns for these markers. AnxA2, S100A10 and the complex were expressed in different types of trophoblasts within the chorionic tissues including syncytiotrophoblasts, cytotrophoblasts (Figs. 3d, 4a, b, d), and different types of invading chorionic trophoblasts (Figs. 4, 5) such as trophoblast giant cells (Fig. 5a–c) and glycogen trophoblasts (Fig. 5d). Perivascular chorionic trophoblasts showed proliferative activities (Fig. 5) with transformation into other trophoblast types such as multinucleated trophoblast giant cells (Fig. 5a–c), glycogen trophoblasts (Fig. 5d). The proximity ligation signal was observed in the same locations (Fig. 5d, e) as the individual proteins, evidence for an in situ complex between the two proteins in these cells. Expression in Hofbauer cells in the chorionic connective stroma was mainly membranal (Fig. 5f).

### Vascular remodelling and expression of AnxA2, S100A10 and the AnxA2/S100A10 complex in the placenta

The placental vasculature is distinctive in that vascular remodelling occurs where the foetal trophoblasts migrate and invade maternal vessels to remodel the vascular wall to form chorionic foetal vessels. During vascular remodelling, perivascular trophoblasts showed various morphological changes such as vacuolisation, elongation and proliferation and trophoblast giant cells and glycogen trophoblasts were frequently seen (Fig. 5a–d). Interestingly, AnxA2 and S100A10 were expressed in trophoblast exhibiting morphological changes characteristic for proliferative activity (Fig. 5a–c). Both proteins appeared to show marked condensation in the cell membrane with distinct vesicular expression (Fig. 5a, c).

**Fig. 6:**
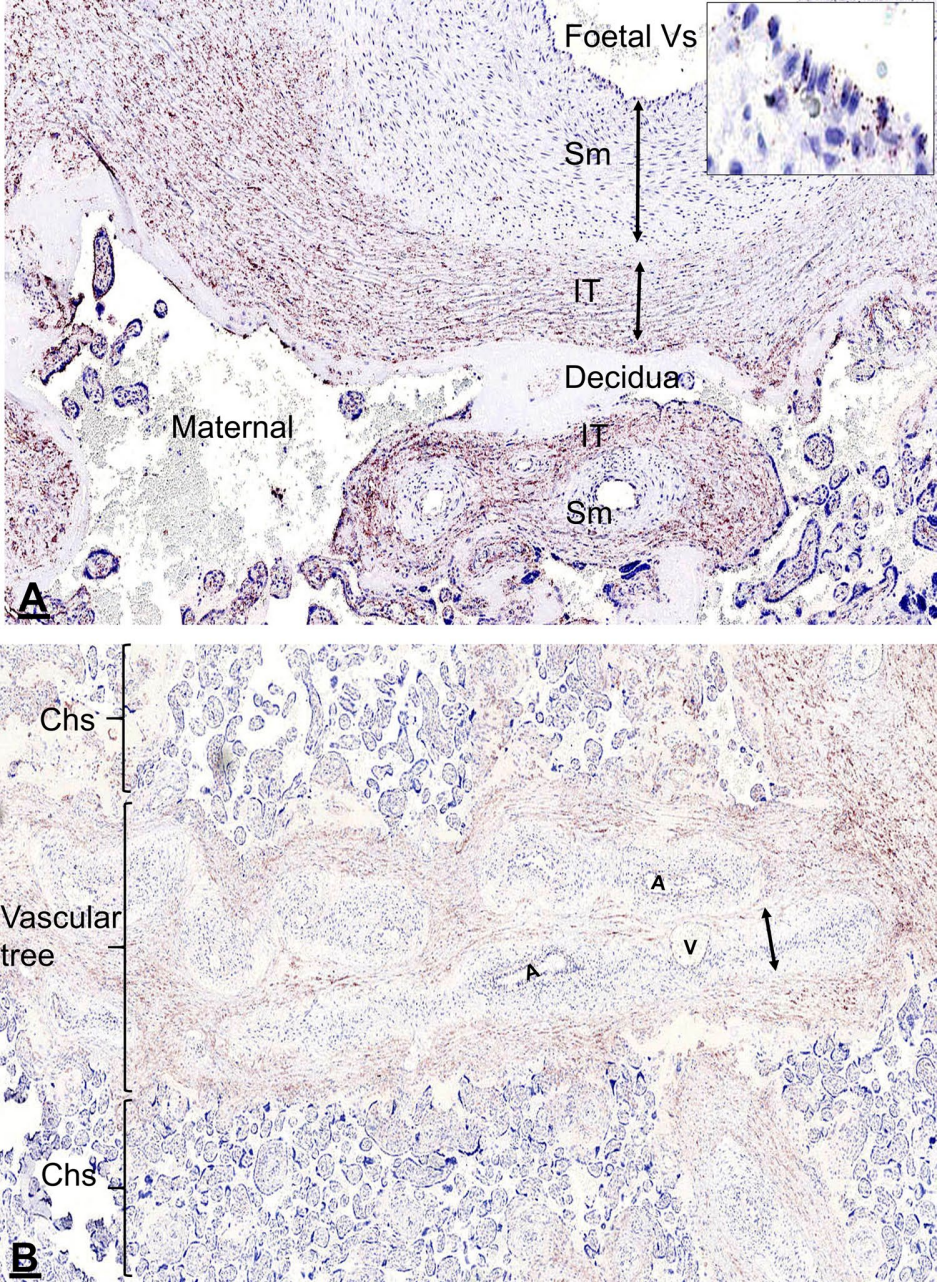
AnxA2/S100A10 complex expression in remodelling placental vessels. **a** A thick wall large size artery (top) and small size foetal arteries (bottom) close to the foetal side (amniotic membrane) showing AnxA2/S100A10 complex expression in the vascular endothelial lining and the interstitial (IT) chorionic tissues surrounding the vessels but not in the vascular smooth muscle layer (Sm). The insert is an enlargement of the endothelial lining showing the complex localisation at the luminal surface. **b** A low magnification central in the placenta to show the overall structure of the placenta; the chorions (Chs) and the vascular tree with blood vessels of different types and sizes including arteries (A) and veins (V) cut at different orientation. Scale bars: **a** 100 µm; **b** 200 µm

**Fig. 7:**
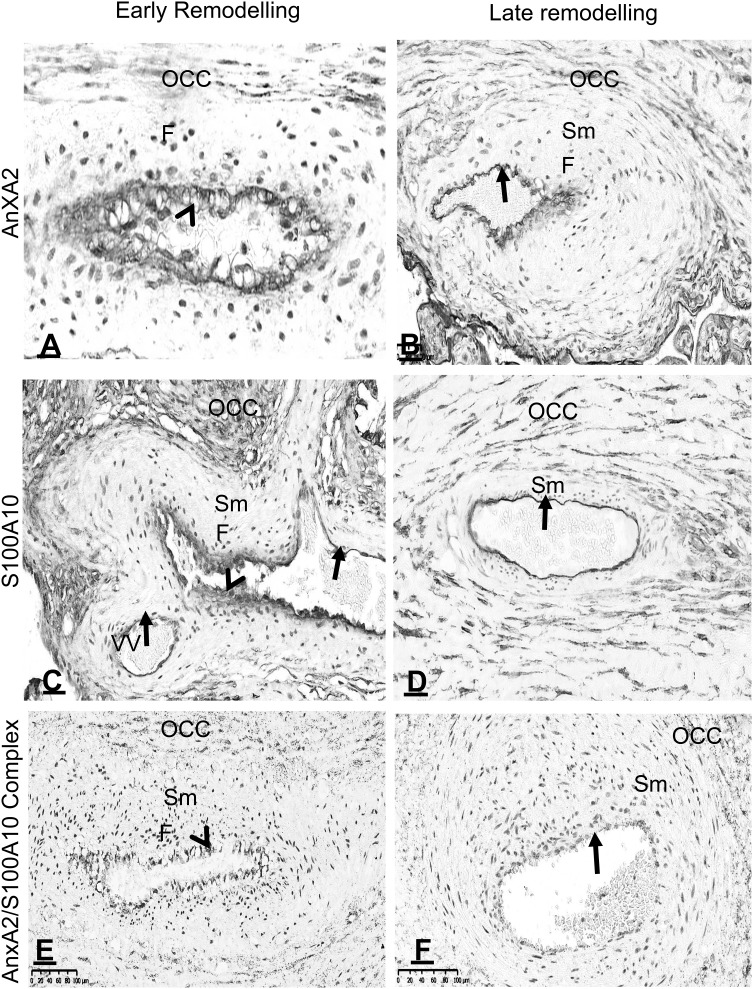
Location of AnxA2, S100A10 and the AnxA2/S100A10 complex in placental vessels at different phases of remodelling. Sections from a placental block containing vessels in early remodelling phase (**a, c, e**) and vessels in late remodelling phase with re-endothelialisation and formation of vascular smooth muscle layer (**b, d, f**) stained for AnxA2 (**a, b**) and S100A10 (**c, d**) are shown. Endothelial lining (arrows), trophoblasts (arrowheads), outer concentric chorionic interstitial tissue (OCC) layers around the vessel, the subintimal fibroid (**f**) deposit, the vascular smooth muscle (Sm) layer and vasa vasorum (VV) are indicated. Scale bars: **a** 20 µm; **b**–**f** 40 µm. Staining is as described in the legend of Fig. 1

Based on the expression of AnxA2, S100A10 and the complex, remodelling changes were seen along the placental vascular tree starting from the large foetal blood vessels (Fig. 6) up to the terminal chorionic foetal vessels (Fig. 4). This included invasion of the perivascular tissues with interstitial trophoblasts (Fig. 5a). Disorganisation of the vessel wall with detachment of the elastic lamina and endovascular trophoblasts incorporation into the vessel wall (Fig. 5b). At advanced phases of remodelling, the original vessel layers were replaced; trophoblasts invaded and replaced the lining endothelial cells and the vascular smooth muscle layer was replaced by interstitial chorionic tissues with sub intimal fibroid deposition (Fig. 7a, c, e). At late phases with completion of the vascular remodelling, there was re-endothelialisation, development of well-defined elastic lamina and vascular smooth muscle layer (Fig. 7b, d, f).

The expression of AnxA2, S100A10 and the complex were studied at different phases of vascular remodelling along the vascular tree. Interestingly, they were constantly localised in the vascular lining through all the vascular tree (Figs. 6, 7). The vascular lining varied according to the stage of remodelling; endothelial cells before remodelling and after completion of the remodelling (Fig. 7b, d, f) and it was invading trophoblasts during the early remodelling phases (Fig. 7a, c, e). AnxA2, S100A10 and the complex were not expressed in the vascular smooth muscle cells all along the vascular tree (Figs. 6, 7). However, this changed during remodelling with trophoblasts invading and replacing vascular smooth muscle layer. Interestingly with completion of remodelling and establishment of the terminal chorionic foetal blood vessels which was usually in the mature chorions, the complex was seen in all vascular layers both in the endothelial lining and the vascular smooth muscle layer (Fig. 4c, d).

## Discussion

In this study we tracked the expression of AnxA2, S100A10 and the complex between these two proteins from the foetal side to the maternal side of the placenta. Studying AnxA2 and S100A10 in parallel, our results confirm and extend earlier studies to show the localisation of both the AnxA2 protein and the S100A10 protein in the placenta. Importantly, we have used the in situ proximity ligation technique to formally investigate the in situ location of the physiologically important complex between the two proteins.

Based on staining of the individual markers, expression of AnxA2 and S100A10 was observed in the amniotic membrane, in blood vessels, in the brush border area of the syncytium and in the trophoblasts. Proximity ligation staining confirmed that the two proteins exist as complexes in these locations. The sensitivity of the proximity ligation staining appeared lower than that of the individual markers therefore the present study largely reveals the locations where the complex is most abundant.

The pattern of AnxA2/S100A10 expression is primarily attributed to the fact that AnxA2 stabilizes S100A10 by protecting S100A10 from being rapidly polyubiquitinated and degraded (He et al. 2008). The amount of S100A10 in the cell would thus dictate the amount of the S100A10/AnxA2 complex in the cell.

AnxA2, S100A10 and the AnxA2/S100A10 complex showed strikingly high expression in the epithelial cells of the amniotic membranes, both at the apical membrane as well as the junctions between cells. Others also showed the presence of AnxA2 and S100A10 in amniotic structures (Bogic et al. 1999; Sun et al. 1996). In the epithelium, the complex may serve several functions. Previous studies in MDCK cells showed that the interaction between AnxA2 and S100A10 provides junctional complexes maintaining the epithelial cell layer (Lee et al. 2004) and it is tempting to speculate that a similar function is served in the amniotic epithelium. The staining pattern at the cell–cell interface would be compatible with such a function. Significant staining of both AnxA2, S100A10 and the AnxA2 /S100A10 complex was observed at the apical membrane, which could be related to secretory and transport processes that are known to occur in this area and which are known to be regulated by the AnxA2/S100A10 complex (Gerke et al. 2005; Mamede et al. 2012). Additionally, high levels of tissue plasminogen activator have been observed at the amniotic epithelium (Bogic et al. 1999; Liu et al. 1998). The AnxA2/S100A10 complex is a receptor for this protein and may regulate this protein locally at the surface of these cells (Madureira et al. 2011).

AnxA2, S100A10 and the complex were expressed in syncytiotrophoblast mostly in the apical membranes and in the brush border. This confirms earlier studies showing the presence of the two proteins in the brush border (Kaczan-Bourgois et al. 1996; Kristoffersen and Matre 1996). An increase in the levels of AnxA2 was accompanied by an increase in the S100A10 in the isolated placental brush border during gestation (Kaczan-Bourgois et al. 1996) and both proteins are located to the cell surface (Kristoffersen and Matre 1996). Our proximity ligation staining indicates the two proteins exist as a complex in this location. However, interestingly, from the relative staining intensities of AnxA2 and S100A10, it appears that some of the AnxA2 protein is not complexed in this location. Previous estimates suggested that on a molar basis, the amount of AnxA2 at this location is about 2–4 times that of S100A10 (Kaczan-Bourgois et al. 1996), and our observations are consistent with that. Thus, a mixture of AnxA2/S100A10 and AnxA2 monomer appears to exist at this location. Both AnxA2 monomer and the AnxA2/S100A10 complex may contribute to membrane-related functions such as exocytosis, endocytosis, membrane reorganization, vesicular trafficking and lipid regulation (Gerke et al. 2005) as well as regulation of ion channels and exchange proteins at this site. The presence of AnxA2 in lipid rafts of the brush border membrane (Paradela et al. 2005) suggests that these functions may be executed at this particular membrane sub-compartment. On endothelial cells, the AnxA2/S100A10 complex has been proposed as surface platform for tissue plasminogen activator and plasminogen, aiding the latter’s conversion to plasmin (Luo and Hajjar 2013; Madureira et al. 2012). As such, cell surface localised AnxA2/S100A10 may be associated with the prevention of fibrin deposits and thrombi in the placenta. Indeed, reduced expression of AnxA2 in pre-eclampsia is associated with increased placental thrombi (Xin et al. 2012).

Expression of the AnxA2, S100A10 and AnxA2/S100A10 complex markers in trophoblasts varied according to the anatomical location, with expression mainly in trophoblasts located in areas with active placentation, such as remodelling vessels and tips of developed chorions, suggesting that the AnxA2/S100A10 complex plays a role in trophoblast invasive activity. To create the placental blood supply, specialized subpopulations of trophoblasts invade through the decidua to engraft and remodel uterine spiral arteries. The complex was expressed in the foetal chorionic trophoblasts. It was present in the vascular lining and outermost vascular boundaries and absent from the vascular smooth muscle cells which are of maternal origin. During remodelling, foetal trophoblasts invade the maternal blood vessel wall and gradually replace the vascular smooth muscle cells (Carter and Pijnenborg 2011; Hunkapiller and Fisher 2008; Red-Horse et al. 2004; Staff et al. 2010; Whitley and Cartwright 2010). The intact maternal vascular smooth muscle cells do not express the complex, however those replaced by trophoblasts did. The complex was present in the chorionic villi but absent from the remnant maternal decidual plate.

Matrix remodelling is an essential step in trophoblast invasion and vascular remodelling in the placenta. A possible role for AnxA2/S100A10 complexes in trophoblast invasion is indicated by silencing of S100A10 as recently reported (Bissonnette et al. 2016). It is compatible with similar functions in other cell types, most notably in cancer cells and endothelial cells (Ling et al. 2004; Surette et al. 2011; Zheng et al. 2011). Genetic deletion of AnxA2 compromises the ability of endothelial cells to activate extracellular matrix proteases, and invade extracellular matrix (Ling et al. 2004) as has genetic deletion of S100A10 (Surette et al. 2011). Cell surface expression of AnxA2 is also required for matrix invasion in neoplastic context. For example, pancreatic cancer cells contributing to a metastatic phenotype (Zheng et al. 2011). As mentioned, the AnxA2/S100A10 complex has been proposed to be a surface platform for tissue plasminogen activator and plasminogen, aiding the conversion to plasmin which in turn directly degrades extracellular matrix components, or activates further extracellular matrix proteases (Luo and Hajjar 2013; Madureira et al. 2012). Interestingly, tissue plasminogen activator has been identified in placental trophoblast cell layers and may be activated by the AnxA2/S100A10 complex to execute invasive functions (Bogic et al. 1999; Liu et al. 1998).

Overall, our study has localised AnxA2/S100A10 complexes to key anatomical locations in the placenta and suggests a role for this complex in amniotic epithelium, trophoblasts and syncytium, in addition to its well-known roles in endothelial cells. Broadly speaking the expression of the complex parallels that of the individual protein partners. However, comparisons between markers in the syncytium indicate the existence of at least two AnxA2 subpopulations at this location, the AnxA2 monomer and the AnxA2/S100A10 complex. Further research is needed to examine the existence of such subpopulations in other locations and cell types under normal and pathophysiological conditions. The proximity ligation technique described here is useful tool to specifically assess the AnxA2/S100A10 complex as marker in normal and diseased tissue and can be powerful to assess the usefulness of other protein complexes as biomarker.
